# Extracellular Vesicles as Potential Prognostic Markers of Lymphatic Dysfunction

**DOI:** 10.3389/fphys.2020.00476

**Published:** 2020-05-25

**Authors:** Andreea Milasan, Maya Farhat, Catherine Martel

**Affiliations:** ^1^Department of Medicine, Faculty of Medicine, Université de Montréal, Montreal, QC, Canada; ^2^Montreal Heart Institute, Montreal, QC, Canada

**Keywords:** lymphatic function, lymph, extracellular vesicles, atherosclerosis, cardiovascular disease, circulating marker

## Abstract

Despite significant efforts made to treat cardiovascular disease (CVD), more than half of cardiovascular events still occur in asymptomatic subjects devoid of traditional risk factors. These observations underscore the need for the identification of new biomarkers for the prevention of atherosclerosis, the main underlying cause of CVD. Extracellular vesicles (EVs) and lymphatic vessel function are emerging targets in this context. EVs are small vesicles released by cells upon activation or death that are present in several biological tissues and fluids, including blood and lymph. They interact with surrounding cells to transfer their cargo, and the complexity of their biological content makes these EVs potential key players in several chronic inflammatory settings. Many studies focused on the interaction of EVs with the most well-known players of atherosclerosis such as the vascular endothelium, smooth muscle cells and monocytes. However, the fate of EVs within the lymphatic network, a crucial route in the mobilization of cholesterol out the artery wall, is not known. In this review, we aim to bring forward evidence that EVs could be at the interplay between lymphatic function and atherosclerosis by summarizing the recent findings on the characterization of EVs in this setting.

## Introduction

In the 1620s, two important networks of vessels were discovered: the blood and the lymphatic circulation (Suy et al., 2016). Whereas tools to study the blood circulation have been developed at a greater pace, exploring the thin and translucent vessels that characterize the lymphatic network had its load of hurdles. Considered as white blood-containing vessels in the early beginnings, the lymphatic system is now recognized as one of the most crucial supporters of the immune response (Jones and Min, 2011). It is generally known to perform three main functions (Cueni and Detmar, 2008). First, the lymphatic system defends the body against infections. The vessels displace lymph throughout the body, and the lymph nodes act as a filter to get rid of debris, bacteria, viruses, and other foreign bodies (Butler et al., 2009). Second, as lymphatic vessels called lacteals are located within the digestive tract, it helps absorb fat-soluble vitamins and dietary fat, which will in turn reach the bloodstream and be used as needed (Iqbal and Hussain, 2009). Third, it is critical in maintaining tissue homeostasis. Excess fluid that escapes from the bloodstream is collected by the lymphatic system, thus preventing the formation of edema (Phelps, 1990).

With the development of genetic mouse models and imaging tools adapted to fit both animals and humans, new functions of the lymphatic system have been brought forward, including the central role of functional adventitial lymphatic vessels the in removal of cholesterol from the atherosclerotic lesion (Lim et al., 2013; Martel et al., 2013; Vuorio et al., 2014; Milasan et al., 2016a, 2019; Rademakers et al., 2017). Atherosclerosis, the major cause of cardiovascular disease (CVD), is characterized by the accumulation of cholesterol and an intensified inflammatory reaction within the artery wall (Libby et al., 2011). The triggered cell activation and apoptosis plays a central role in the disease progression and results in the formation and accumulation of submicron particles called extracellular vesicles (EVs) within the blood vessel intima (Leroyer et al., 2007; Bobryshev et al., 2013). As EVs interact with surrounding cells to transfer their cargo that comprises messengerRNA (mRNA), microRNA (miRNA), proteins and lipids, they are suspected to be key players in the exacerbation of atherosclerosis. The lymphatic network is a crucial route in the mobilization of cholesterol out the artery wall (Martel et al., 2013), and we hypothesize that the massive accumulation of EVs in the artery wall could also be due to a poor clearance by the lymphatic vessels located in the affected blood vessels. Albeit the advances in analytical technologies combined with improved lymph collection techniques have led to the detection of EVs in mouse lymph (Milasan et al., 2016b), the fate of EVs within the lymphatic vessels is only emerging. In this review article, we discuss the association between lymphatic function and CVD, provide an overview of the biogenesis, functions and role of EVs in atherosclerosis, and bring forward evidence of a causal relationship between EVs and lymphatic dysfunction associated with atherosclerosis.

## The Role of the Lymphatic System in Atherosclerosis

Hoggan and Hoggan (1882) reported that lymphatics are present in the arterial wall. A century later, Dr. Gerald Lemole suggested that the accumulation of interstitial fluid in the artery wall may contribute to the development of atherosclerosis due to factors present in the intimal edema, and instigated the concept of lymphstasis in atherogenesis (Lemole, 1981). The following decade revealed that disruption of cardiac lymphatic drainage in allogenic transplanted hearts could be at the origin of the enhanced atherosclerosis observed (Miller et al., 1992). Lymphatic vessels are present in the adventitial layer of atherosclerotic arteries (Kholova et al., 2011), and Martel et al. (2013) have reported that functional adventitial lymphatic vessels are essential to first mobilize cholesterol out of the vessel wall before it reaches the thoracic lymphatic duct and the blood circulation at the level of the subclavian vein, bringing front stage the role of lymphatics in macrophage reverse cholesterol transport (mRCT). Using a surgical model of aortic transplant from a hypercholesterolemic *apoe^–/–^* donor mouse to a hypercholesterolemic *apoe^–/–^* receiver mouse in which an apoE vector was subsequently injected to induce cholesterol efflux, the authors revealed that the newly formed lymphatic vessels facilitated cholesterol removal from advanced plaque (Martel et al., 2013). Mice that were given an anti-VEGFR3 antibody to prevent the development of lymphatic connections between the transplanted aorta and the receiver’s artery had enhanced cholesterol accumulation compared to the control mice when the apoE vector was given. Subsequently, it had been shown that treatments known to reduce lipid and immune cell accumulation within the aortic root of hypercholesterolemic mice (Wilhelm et al., 2010) were potentially mediating their beneficial effects through the enhancement of lymphatic function (Milasan et al., 2017). In their manuscript, Milasan and collaborators injected lipid-free apoA-I intradermally in mice bearing mature atherosclerosis lesion and observed that the reduction in plaque size was associated to an improved molecular and cellular lymphatic transport and to a significant drop in the atherosclerosis-associated collecting lymphatic vessel leakage. ApoA-I appeared to strength junctions between lymphatic endothelial cells (LECs) through an upregulation of the VEGFR-3 pathway in LECs. Furthermore, *ex vivo* experiments revealed that apoA-I also acts upon the enhancement of platelet adhesion on the lymphatic endothelium and on the reduction of platelet aggregation induced by either thrombin or podoplanin (Milasan et al., 2017). The authors envisioned that by limiting platelet aggregation, apoA-I would clear the way for platelet adhesion on LECs, which would in turn exert a shielding effect on the lymphatic endothelium, just like macrophages are exercising a “bridge effect” between adjacent blood endothelial cells (EC) (He et al., 2016). By enhancing the adherence of pseudopodia-shaped platelets that able to reach and pull several LECs together, apoA-I might reinforce the lymphatic endothelial barrier and thus contribute to the preservation of the lymphatic endothelium integrity in atherosclerotic subjects.

The prerequisite role of the lymphatic system in onset of atherosclerosis was demonstrated using atherosclerosis-prone mice (*ldlr^–/–^; hapoB100^+/+^*) (Milasan et al., 2016a). At 3 months of age, these mice displayed an impaired lymphatic function, even before the onset of atherosclerosis. This early dysfunction that was not cholesterol-dependant was associated to a defect in the collecting lymphatic vessels, rather than a defect in the absorptive capacity of the initial lymphatic vessels (also called lymphatic capillaries) (Milasan et al., 2016a). Collecting vessels are contractile lymphatics that propel lymph in a unidirectional manner, with the help of intraluminal bi-leaflet valves and lymphatic muscle cells (LMCs) that cover sporadically the functional pumping unit of a collecting lymphatic vessel called the lymphangion (Zawieja, 2009). LMCs enable the spontaneous contraction of the lymphatics. The follow-up study reported that early treatment with a mutant form of VEGF-C (VEGF-C152S), a selective agonist of VEGFR-3, rescues the contractile capacity of collecting lymphatic vessels, delays plaque onset and limits its progression (Milasan et al., 2019). Whereas the exact mechanisms responsible for the prompt defect in lymphatic function observed prior to the atherosclerosis plaque onset remain to be elucidated, these findings strongly suggest that targeting lymphatic function in patients at risk of developing coronary artery disease (CAD) may constitute a novel therapeutic target for the prevention and treatment of atherosclerosis.

## The Diversity of Extracellular Vesicles Drives Their Fate and Function in Atherosclerosis

EVs are released by cells under physiological and pathological conditions and express markers pertaining to their cell of origin (Yuana et al., 2013). Due to their diversity and prominent presence in tissues and fluids all over the body (Freyssinet, 2003), they are considered important potential markers in health and disease, including in rheumatoid arthritis (Boilard et al., 2010; Fu et al., 2018), tumor progression (O’Loghlen, 2018), angiogenesis (Todorova et al., 2017), metastasis (Peinado et al., 2017; Zhao et al., 2018), diabetes (Freeman et al., 2018), hypertension (Manakeng et al., 2019), metabolic syndrome (Diamant et al., 2002; Martinez and Andriantsitohaina, 2017), hypercholesterolemia and CVD (Jansen et al., 2017b). EVs that are derived from diverse cell types such as leukocytes, erythrocytes, smooth muscle cells (SMCs) and endothelial cells (ECs), are found in atherosclerotic lesions as a result of cell activation or death (Leroyer et al., 2007). Whereas red blood cell (RBC)- and platelet-derived EVs are the most abundant EV subsets in the blood vasculature, platelets, leukocytes and vascular cells are also known to release EVs in the circulation. Notwithstanding, CVD is associated to increased concentrations of EVs, regardless of the cell of origin. Numerous studies have explored and confirmed that circulating and tissue EVs originating from diverse cell types exert different roles in key steps of atherosclerosis and the subsequent clinical outcomes (reviewed in Boulanger et al., 2017). All EVs are surrounded by a lipid bilayer like that of a cell plasma membrane, in contrast to the single-layered high-density lipoprotein (HDL) and low-density lipoprotein (LDL) (Boulanger et al., 2017). However, the cargo they transfer, namely proteins, lipids, miRNAs, non-coding RNAs and surface receptors and antigens, depends on the cell they originate from Diamant et al. (2004) and therefore alters the functional state of the recipient cells in a different manner (Silva and Melo, 2015). In addition of differing in morphology, cellular origin, number, antigenic composition and functional properties, EVs are also heterogenous in size, even if they are originating from the same cell type (Boulanger et al., 2017).

EVs are classified according to their size and mechanism of formation, and it is possible to distinguish these diverse released populations: exosomes, exocytosed from multivesicular bodies; microvesicles (MVs) that bud directly from the plasma membrane; and apoptotic bodies which result from apoptotic blebbing following cell death (Raposo and Stoorvogel, 2013). The release of EVs is a process conserved through evolution, which indicates how essential their role is physiologically (Boilard et al., 2015). To better understand the impact of the release of each subset in CVD, we will herein explore in greater details these submicron vesicles individually.

### Exosomes

Exosomes constitute the smallest subpopulation of EVs with a size ranging between 40 and 120 nanometers (nm) (Zaborowski et al., 2015). Secreted by various types of cells, exosomes are formed from multivesicular bodies (MVB), specialized endosomes containing intraluminal vesicles (Hessvik and Llorente, 2018). The MVB are involved in several functions related to endocytosis and protein trafficking such as sorting, recycling, transport, storage and release (Borges et al., 2013; Shao et al., 2018). Exosome formation can be divided into several stages. First, microdomains rich in lipids and membrane-associated proteins are formed on the membrane limiting the MVB. In this process, cytosolic cargo destined for inclusion in EVs are recruited to the microdomains (van Niel et al., 2018). Consequently, intraluminal vesicles are generated by inward budding from the endosomal membrane into the MVB lumen. These MVB can thereafter fuse with the plasma membrane and be released into the extracellular medium to form exosomes (Raposo and Stoorvogel, 2013). Exosome biogenesis involves different molecular mechanisms and can be either dependent or independent of the endosomal sorting complex required for transport (ESCRT) (van Niel et al., 2018).

Given their origin, exosomes contain endosome-associated proteins, such as GTPase Rab, SNAREs, Annexins and flotillin (Raposo and Stoorvogel, 2013). They also harbor a set of evolutionally conserved proteins, including Tsg101 and Alix, and are rich in lipids, such as cholesterol and ganglioside GM121 (Borges et al., 2013; Osteikoetxea et al., 2015; Kowal et al., 2016). Additionally, they contain heat shock proteins HSP60, HSP70, HSPA5, CCT2, and HSP90 (Hong et al., 2018). Tetraspanins, such as CD9, CD63, and CD81 are the most frequently identified proteins and by now, they are considered good general markers of exosomes (Andreu and Yanez-Mo, 2014). However, they may be expressed by other subtypes of EVs emerging from the plasma membrane due to their presence on the surface of various cell types and have already been identified on the surface of MVs as well (Willms et al., 2018). Exosomes contain more sphingomyelins, gangliosides, and desaturated lipids, while their phosphatidylcholine and diacylglycerol proportion is decreased when compared to the membranes of the cells from which they originate (Laulagnier et al., 2004). Furthermore, they are enriched with nucleic acids, such as mRNA and miRNA, further supporting the hypothesis that they are a biological vehicle with the ability to modulate protein synthesis of the target cell and can confer it new functions (Valadi et al., 2007). Exosomes have been identified in several body fluids, such as plasma, urine, saliva, bile, breast milk, sperm, amniotic fluid, cerebrospinal fluid, ascites fluid (reviewed in Yanez-Mo et al., 2015) and most recently, in lymph (Milasan et al., 2016b). They can be released by practically any cell type following activation or apoptosis, including ECs, platelets, RBCs, SMCs, dendritic cells, monocytes and macrophages, and cardiomyocytes (Golchin et al., 2018). Importantly, the microenvironment features, such as hypoxia, also exert important effects on the properties of the origin cell-derived exosomes (Willms et al., 2018). Exosomes produced by cells exposed to oxidative stress can mediate protective signals, thus reducing oxidative stress and cell death in recipient cells (Eldh et al., 2010). Exosomes can also contain cytokines that induce inflammation via numerous different pathways (Distler et al., 2005), and can contribute to cell aggregation following neutrophil and leukocyte recruitment (Camussi et al., 2010). Therefore, exosomes, and especially their cargoes, play different key roles in various normal physiological instances and pathological responses to disease.

### Microvesicles

Microvesicles bud directly from the plasma membrane, measure approximately 100 nm to 1 μm in size and contain cytoplasmic cargo (Zaborowski et al., 2015). They are formed by the remodeling of the cytoskeleton, and their release is increased under inflammatory conditions, hypoxia or activation (Morel et al., 2011; Boulanger et al., 2017). Following cell stimulation, a cytosolic influx of calcium ions (Ca^2+^) may disrupt the asymmetric distribution of phospholipids in the membrane bilayer by activation of the scramblase involved in the translocation of membrane phospholipids. This results in redistribution of phospholipids. Subsequently, this leads to phosphatidylserine (PS) translocation, creating an imbalance between the internal and external leaflets that leads to budding of the plasma membrane and MVs release. The cytoskeleton degradation caused by the Ca^2+^-dependent proteolysis promotes the budding of these vesicles which can then express at their surface phospholipids such as PS, that normally constitute the inner membrane layer (Hugel et al., 2005). This translocation can then be used to identify them (Shao et al., 2018). However, it has already been observed in plasma that not all MVs externalize PS (Arraud et al., 2014; Boulanger et al., 2017), but they contain flotillin-2, selectins, integrins, and metalloproteinases (Borges et al., 2013). Highly discussed is the fact that circulating vesicles seem to be composed of both exosomes and MVs. Therefore, currently available purification methods do not yet allow to fully discriminate beyond a reasonable doubt between these two entities.

### Apoptotic Bodies

Apoptotic bodies are released as a result of apoptotic cell disassembly and consist of apoptotic material surrounded by a permeable membrane. Apoptotic bodies typically range in size between 1 and 5 μm (Atkin-Smith and Poon, 2017), under certain conditions can become more abundant than exosomes and MVs, and vary greatly in content between biofluids (El Andaloussi et al., 2013). Furthermore, the rapid clearance of apoptotic body fluids by phagocytic cells complicates their characterization (Pitt et al., 2016).

Although exosomes and MVs have been more thoroughly studied to date, as they are significantly and differentially involved in diverse pathologies, apoptotic bodies have similar functional importance with respect to immunomodulatory effects. Exosomes represent an attractive mean of cargo transportation, and many studies to date focused on understanding the precise functions of these smaller entities. Interestingly, apoptotic cells are also suspected to release exosomes, but it remains to be confirmed (Caruso and Poon, 2018). Apoptotic bodies are created to aid in apoptotic cell clearance, as well as a means of intercellular communication. They are involved in the horizontal transfer of DNA including tumor DNA that can result in the induction of a tumorigenic phenotype, in the presentation of epitopes to T cells via internalization by phagocytic cells and in the presentation of autoantigens to B lymphocytes (Bergsmedh et al., 2001; Gyorgy et al., 2011). Apoptosis is an important process in different immunological disease settings such as inflammation, infection, autoimmunity, and cancer (Zitvogel et al., 2010; Hochreiter-Hufford and Ravichandran, 2013; Poon et al., 2014).

## Internalization of Extracellular Vesicles

Atherosclerosis onset and progression is associated with the accumulation of several subsets of EVs that are internalized differently and thus harbor distinct functions in several stages of the disease. An abundance of mechanisms can be involved in the internalization of EVs inside target cells (Borges et al., 2013). Currently, an increasing number of specific protein-protein interactions that seem to differentially mediate EVs uptake/internalization are on the rise (Mulcahy et al., 2014). Tetraspanins are abundantly expressed by EVs in raft-like structures within their plasma membrane and are generally involved in adhesion, displacement, fusion, activation and proliferation (Hemler, 2005; Andreu and Yanez-Mo, 2014). Inhibition of tetraspanin expression with antibodies was shown to decrease the internalization of EVs in target cells (Rana et al., 2012). Integrins and immunoglobulins, involved in leukocyte adhesion and transmigration, as well as intracellular signaling, also seem to be involved in EVs uptake (Mulcahy et al., 2014). Blockade of CD11a, its ligand ICAM-1, CD51 and CD61 each caused a decrease in the internalization of EVs by dendritic cells (Morelli et al., 2004a; Mulcahy et al., 2014). Proteoglycans are entities that contain a significant amount of carbohydrate components, such as heparin sulfate proteoglycans. Treating bladder cancer cells with a heparin sulfate mimetic reduced cancer cell exosomes uptake thus showcasing their role as key receptors of macromolecular cargo (Christianson et al., 2013; Franzen et al., 2014). Lectins such as DC-SIGN, DEC-205 and Galectin-5, which can trigger phagocytic entry, have also been associated with the internalization of EVs (Hao et al., 2007; Blanc and Vidal, 2010; Naslund et al., 2014).

Endocytosis is another mechanism largely involved in the internalization of EVs. Uptake of EVs within the endosome can occur in a matter of minutes and this internalization was significantly reduced at 4°C, which demonstrates that endocytosis of EVs is an active process that requires energy (Morelli et al., 2004b; Mulcahy et al., 2014). Cytochalasin D, an actin depolymerizing agent that alters endocytosis in mammalian cells, has also contributed to a decrease in EVs internalization in several cell types (Mulcahy et al., 2014). Clathrin-mediated endocytosis involves the progressive formation of clathrin-coated vesicles expressing several transmembrane receptors, as well as their ligands. This allows them to integrate the target cell, undergo clathrin un-coating and fuse with the endosome where it can release its contents (Kaksonen and Roux, 2018). Alternatively, caveolin-dependent endocytosis involves the invagination of caveolae, which are subdomains of lipid rafts, formed by the action of caveolin (Doherty and McMahon, 2009). Several studies demonstrate that both types of endocytosis are involved in the internalization of EVs. Blocking dynamin 2, a highly conserved GTPase involved in endocytosis and vesicle transport, significantly impaired exosome entry into cells (Yao et al., 2018). Since dynamin 2 is relied upon by both types of endocytosis, further confirmation of caveolin-dependent endocytosis-specific implication requires knockdown of the *Cav1* gene (Nanbo et al., 2013). EVs can also be internalized via phagocytosis which is induced by physical contact with receptors on the surface of specialized phagocytic cells, such as macrophages (Zent and Elliott, 2017). The use of wortmannin and LY294002, PI3-kinase inhibitors that prevent phagosome formation, caused dose-dependent inhibition of the internalization of exosomes within macrophages (Feng et al., 2010; Mulcahy et al., 2014). PS, important in phagocytosis of apoptotic bodies, is frequently externalized on EVs outer membrane (Fomina et al., 2003) and seems to be involved in their internalization. Treatment with inhibitors that bind TIM4, present on macrophages and involved in PS-dependent phagocytosis, or that directly bind PS, such as annexinV, significantly reduced EVs uptake within macrophages and natural killer cells (Nolte-’t Hoen et al., 2009; Feng et al., 2010; Yuyama et al., 2012).

Recently, EVs were shown to depend mainly on macropinocytosis and clathrin-independent endocytosis to enter cells (Costa Verdera et al., 2017). Macropinocytosis involves the invagination of the cell membrane ruffles and its retraction into the intracellular compartment (Mulcahy et al., 2014). Clathrin-independent endocytosis, alternatively called raft-dependant endocytosis, requires functional lipid rafts within the plasma membrane and depends on cholesterol (Teissier and Pecheur, 2007). Lipid rafts are found within invaginations formed by caveolin-1 or in planar regions of the plasma membrane that associate with flotillins (Hooper, 1999). As cholesterol reducing agents like filipin and simvastatin have been shown to prevent EVs uptake, lipid rafts are suspected to play a role in EVs internalization (Costa Verdera et al., 2017; Pfrieger and Vitale, 2018). Furthermore, inhibition of lipid rafts prevented the release of platelet-derived EVs (PEVs) that expose PS at their surface (Mulcahy et al., 2014; Wei et al., 2018). Lastly, EVs membranes can directly fuse with the cell plasma membrane. Increased exosome uptake at low pH by fusion with melanoma cells was observed *in vitro* potentially due to EVs lipid content and ionic charge, as assessed by pre-treatment with proton pump inhibitors (Parolini et al., 2009).

Since the precise proportions of each mechanism involved in EVs internalization are incompletely defined, the consensus is that endocytosis is primarily involved through surface binding. Overall, studies suggest that despite the fact that EVs can be taken up by virtually every cell, through a variety of mechanisms, specificity to certain target cells is not to be neglected, as the engulfment of certain EVs is more effective in one cell type than another (Mulcahy et al., 2014).

## The Role of Lymph Extracellular Vesicles in Intercellular Communication

Beyond classical signaling through cell–cell contact and soluble factors, such as cytokines, inflammatory mediators, metabolites, and hormones, EVs are now recognized by the scientific community as important mediators of both local and systemic cellular communication (Huang-Doran et al., 2017; Hutcheson and Aikawa, 2018). EVs have several functions depending on the number of bioactive molecules, surface receptors, and genetic information they carry, as well as the cell type of origin and the particular physiological and pathological condition at the time of their packaging and secretion (Montecalvo et al., 2012). Their membrane bilayer gives them the ability to protect their cargo from the enzymes that could degrade them, such as ribonuclease or trypsin (Koga et al., 2011). It has been demonstrated that the mRNAs and miRNAs contained within EVs, once transferred to target cells, are translated into proteins, or regulate gene expression via *de novo* translation or post-transcriptional regulation (Valadi et al., 2007), and may even favor specific signaling cascades in order to induce phenotypic changes (Al-Mayah et al., 2012). Several examples showcase how EVs can differentially control the function of specific cell types, including types distinct of those from which they originated (Borges et al., 2013).

The critical role of plasma EVs derived from platelets, leukocytes, ECs and others in activating immune, endothelial and vascular SMCs, have been extensively described (reviewed in Oggero et al., 2019). However, studies decrypting the role of the lymphatic circulation in the transport of EVs are only starting to emerge. New insights are available into how lymphatics could contribute to the clearance of these inflammatory mediators and support their role in cell-cell communication. The inflammatory response occurring after trauma/hemorrhagic shock (T/HS) is now believed to be driven by the affluence of mesenteric lymph. In a study conducted in rats that underwent T/HS, Kojima et al. (2017) demonstrated that the exosomes contained in mesenteric lymph are the major component triggering inflammatory responses in monocytes and macrophages after T/HS. Cancer-related studies also revealed that exosomes, herein derived from melanoma, can travel through the lymphatic vessels to accumulate in the lymph nodes and promote tumor metastasis (Hood et al., 2011). These nanosized vesicles can be transported by the lymphatic vessels from peripheral tissues to draining lymph nodes where they can be observed for up to 2 days. The internalization of exosomes by macrophages and B cells appears to play an important role in this process (Srinivasan et al., 2016). A study confirmed in mice revealed that postoperative lymphatic exudate of metastatic melanoma patients provides a rich source of extracellular vesicles containing melanoma-associated proteins and miRNAs enabling the differentiation between early and advanced metastatic spread (Broggi et al., 2019). Similarly, Maus et al. (2019) reported that lymph EVs aid in pre-metastatic niche formation in sentinel lymph nodes in human. Earlier this year, Tessandier et al. (2020) unraveled the route followed by EVs that are accumulating in the synovial fluid during rheumatoid arthritis (RA) and identified lymph as the main path involved in the drainage of EV from an inflamed joint. The group thus hypothesized that the transport of the EVs in lymph during RA may represent a way for platelets to transfer their cargo to tissue locations outside blood vessels. Albeit the exact fate of these PEVs in the context of RA remain unstudied, these observations may be transposable to other vascular inflammatory conditions in which platelets play a major role.

As of today, most of the effects mediated through the internalization of EVs has been thought to be caused by the transfer of their protein and nucleic acid components. Lipids have been implicated in multiple aspects of EV biogenesis and function. Studies aiming at characterizing the EV lipidome should be insightful to further understand the role of EVs in several disease onset and progression (Ouweneel et al., 2019).

## Extracellular Vesicles Are Associated With Several Stages of Atherosclerosis

EVs are thought to contribute to vascular endothelial dysfunction, which is considered an early stage of atherosclerosis. For instance, studies have reported that EVs can alter the protective function of the vascular endothelium by interfering with nitric oxide (NO) synthesis (Amabile et al., 2005; Agouni et al., 2008) or by directly increasing its permeability (Densmore et al., 2006). EVs are also believed to play an active role in plaque destabilization, as they can contain various proteolytic factors that promote matrix degradation (van der Vorst et al., 2018). Treatment with EVs derived from human atherosclerotic lesions enhanced EC proliferation *in vitro* and induced angiogenesis *in vivo* (Jansen et al., 2017a). These effects were more pronounced when EVs were isolated from the plaques of symptomatic patients rather than those without any symptoms, pointing out an important determinant of plaque vulnerability (Leroyer et al., 2008). Whereas still debatable, other studies report that the different subsets of EVs display distinct effects on thrombosis. For instance, in human atherosclerotic lesions, the presence of exosomes appear to have antithrombotic effects (van der Vorst et al., 2018) while MVs have been associated with thrombogenic effects (Leroyer et al., 2007). Along with tissue factor (TF) and exposure of PS on the outer membrane layer, MVs are suspected by these teams to contribute to the coagulation pathway (Mallat et al., 1999; Boulanger et al., 2017).

Another study showed that patients with stable CAD but high levels of circulating CD31^+^/annexinV^+^ EVs were at higher risk for coronary revascularization and cerebral events (Sinning et al., 2011). Patients that are at high risk of developing a major cardiovascular event have been shown to have higher levels of CD3^+^/CD45^+^ EVs and SMA-α^+^ EVs (Chiva-Blanch et al., 2016). In a group of patients with diverse cardiovascular risk factors for CAD, levels of circulating CD144^+^ EC-derived EVs, measured by flow cytometry as an indicator of endothelial dysfunction, predicted future cardiovascular events (Nozaki et al., 2009). The presence of subclinical atherosclerosis in asymptomatic patients was shown to be associated with increased levels of leukocyte derived MVs (Chironi et al., 2006). In patients with acute stroke, the levels of EC-derived EVs correlated with lesion volume and clinical outcome (Simak et al., 2006). The accumulation of EVs within the artery wall can undoubtedly be detrimental, raising the need for a better control of EVs homeostasis.

## Lymph Extracellular Vesicles and Their Potential Contribution to Atherosclerosis

**FIGURE 1:**
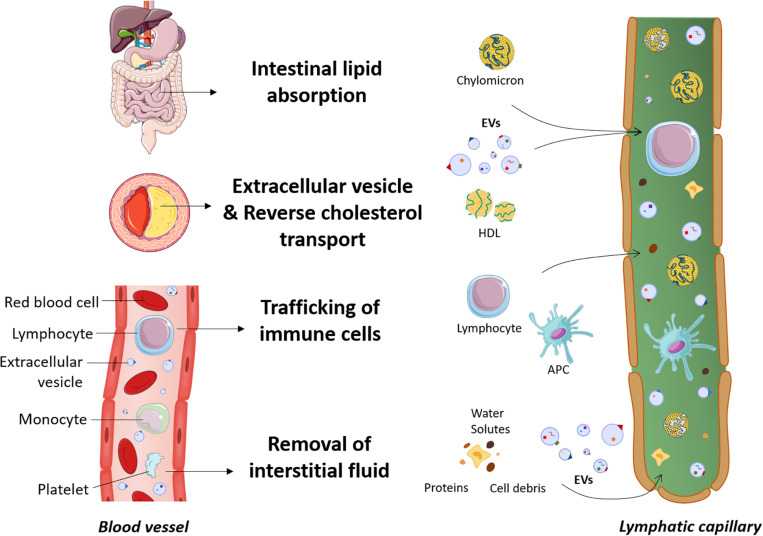
New insights into the roles of the lymphatic system. The lymphatic system is involved in dietary lipid absorption, preservation of fluid balance and host defense. In the past years, more far-reaching effects on several diseases, including cardiovascular disease, have been discovered. Lymphatic vessels are now known to play a prerequisite role in macrophage reverse cholesterol transport. Cholesterol is transported by cholesterol acceptors such as HDL particles through the adventitial lymphatics before reaching the bloodstream. As extracellular vesicles are also a constituent of the atherosclerotic lesion, we hypothesize that these cell fragments also preferentially travel through the lymphatics to be mobilized out of the artery wall. Extracellular vesicles are abundant in the blood circulation, and since plasma ultrafiltrates are collected by the lymphatic system after escaping from the bloodstream, we envision that extracellular vesicles could easily access the lymphatic circulation along with proteins, cells debris and other macromolecules. APC, antigen presenting cells; EVs, extracellular vesicles; HDL, high-density lipoprotein.

Clearance of EVs is thought to occur through different mechanisms that include annexin V-PS receptor-dependent mechanisms (Jansen et al., 2012), phagocytosis by splenocytes (Al Faraj et al., 2012), internalization by endothelial cells, macrophages (Dasgupta et al., 2009, 2012; Imai et al., 2015) or Kupffer cells (Willekens et al., 2005). Nevertheless, our understanding of the mechanisms underlying the clearance of EVs from the peripheral tissues such as the atherosclerotic lesion, the interstitial space or the blood circulation is still in an embryonic stage. Figure 1 depicts the new insights described herein on the different roles of the lymphatic system, including the dissemination of EVs.

**FIGURE 2:**
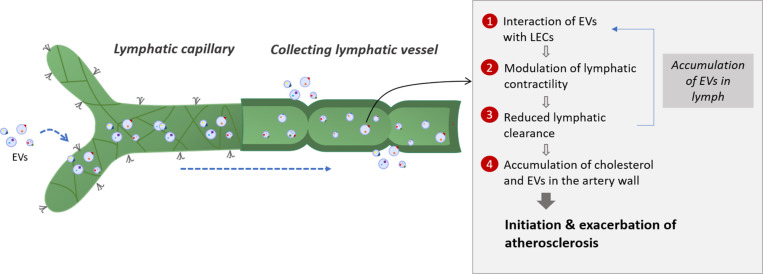
Potential interplay between lymphatic function and extracellular vesicles in atherosclerosis. Extracellular vesicles are first taken up by lymphatic capillaries and then transported through the lymph. Once they reach the collecting lymphatic vessels, extracellular vesicles are thought to modulate the lymphatic contractility by interacting with lymphatic endothelial cells (LECs). Adipose EVs could also interact directly with the collecting lymphatic vessels by affecting the lymphatic muscle or endothelial cells integrity. The potential reduced lymphatic clearance could then result in the accumulation of extracellular vesicles in the lymphatic vessels which will enhance the effect of extracellular vesicles on LECs, thus creating a feedback loop. A defect in collecting lymphatic vessels could also mirror a decrease in the reverse cholesterol transport from the artery wall and promote plaque build-up. Several subsets of extracellular vesicles will then accumulate in the atherosclerotic lesion due to a poor clearance by the adventitial lymphatic vessels, thus modulating the disease progression according to the EVs subsets involved. EVs, extracellular vesicles; LEC, lymphatic endothelial cell.

Plasma is continuously filtered to the extracellular space by a semipermeable layer of blood endothelial cells. The majority of the extravasated interstitial fluid and macromolecules are reabsorbed by the lymphatic capillaries, whereas venules are responsible for transient reabsorption (Levick and Michel, 2010; Aspelund et al., 2016). Overall, an average of 3 l of plasma extravasates from the blood circulation every 9 h and is returned in its majority to the systemic circulation by the lymphatic system (Levick and Michel, 2010). Considering the crucial role of the lymphatic system in the clearance of cells and molecules from peripheral tissues, it is not surprising that circulating EVs can also be found in lymph. A study by our group reported for the first time the presence of PEVs and RBC-derived EVs, inclusively but not exclusively, in the lymph of healthy mice and at higher concentrations in atherosclerotic mice (Milasan et al., 2016b). EVs can thus easily travel from the plasma ultrafiltrate to the lymphatic circulation through initial lymphatics. Additionally, our preliminary data suggest that EVs could act on LECs, modulate their function and alter lymphatic vessel integrity (Jean et al., 2018). These observations lead us to believe that EVs could initially be absorbed adequately by initial lymphatics, while subsequently reaching and affecting the contractile capacity of collecting vessels (Figure 2).

Whereas our data suggest that EVs circulating within the lymphatic vessels are responsible for the various effects on the lymphatic endothelium, we do not exclude that EVs contained in the surrounding environment of the lymphatics can also affect the lymphatic contraction capacity. Collecting lymphatic vessels are generally embedded in adipose tissue (Harvey, 2008; Escobedo and Oliver, 2017), such as in the heart (epicardial adipose tissue) (Montani et al., 2004), around the aorta (Martel et al., 2013), in the skin (Tavakkolizadeh et al., 2001) or in the intestines (Bernier-Latmani et al., 2015). EVs that are derived from various cell types, such as adipocytes (Durcin et al., 2017; Clement et al., 2020) or macrophages (Ying et al., 2017) have been found in peripheral fat and associated with inflammation (Wadey et al., 2019; Dini et al., 2020). Fat and the immune cells it contains are known to affect lymphatic function (Kuan et al., 2015; Escobedo and Oliver, 2017). We thus also suggest that adipose EVs derived from these cells upon activation or death might in turn be able to interact directly with the collecting lymphatic vessels by transferring their cargo to lymphatic muscle or endothelial cells and thus modulate their integrity and affect lymph transport. Regardless of how EVs would get in lymph, they could disturb lymphatic function and therefore modify lymph flow. This could result in the accumulation of EVs within the lymphatic vessels, which would enhance the effect of EVs on LECs and create a feedback loop (Figure 2). Altogether, these sequential events would potentially contribute to the instigation of the lymphatic transport impairment that precedes the onset of atherosclerosis (Milasan et al., 2016a).

Since cholesterol preferentially travel through the lymphatic vessels to get out of the artery wall (Martel et al., 2013), a defect in collecting lymphatic vessels could mirror a decrease in cholesterol transport and promote plaque build-up. Furthermore, the massive accumulation of EVs in the atherosclerotic lesion could also be due to a poor clearance by the adventitial lymphatic, favoring disease progression and exacerbation. Based on these findings, EVs can potentially be considered as the missing link between atherosclerosis and lymphatic dysfunction. However, whether and how specific subsets of EVs could control lymphatic function requires further attention (Raposo and Stoorvogel, 2013; van Niel et al., 2018).

## Potential Role of Extracellular Vesicles on Lymphatic Function

As mentioned before, LECs are exposed to the cargo of numerous subsets of EVs. But what is the direct effect of lymph EVs on lymphatic endothelial and muscle cells? To better understand whether and how EVs might affect lymphatic vessel function, a thorough review of the fate of EVs on the blood endothelium is requisite. We will herein focus on subsets that are also confirmed to be found abundantly in lymph (Milasan et al., 2016b).

### Extracellular Vesicles Released by Red Blood Cells

Red blood cells are found in the largest quantity in the blood, accounting for nearly 83% of total cells (Nemkov et al., 2018), and are one of the main vesicle-secreting cells in the blood circulation (Harisa et al., 2017) as they shed most of their damaged content by vesiculation to prolong their lifespan (Leal et al., 2018). EVs derived from RBCs exert a procoagulant activity as they can generate thrombin by a factor XIIa126-dependent mechanism and stimulate thrombus formation or erosion of the atherosclerotic plaque in proportion to their exposure to TF (Biro et al., 2003; Leroyer et al., 2007). The presence of PS on the surface of RBC-derived EVs provides a site for the assembly of prothrombinase promoting a thrombin clot. Additionally, PS-exposed RBC-derived EVs provide sites for adhesion of platelets and neutrophils localized in the subendothelium, further aggravating CVD progression (Noh et al., 2010).

The main function of RBCs is the transport of oxygen from the lungs to body tissues, and of CO_2_ as a waste product, away from tissues and back to the lungs (Harisa et al., 2017). RBCs are also recognized to supply ATP, which in turn contributes to vessel dilation by stimulating NO production in the endothelium (Bakhtiari et al., 2012). They also typically contain enzymes and molecules with antioxidant activities (Harisa et al., 2017). Although RBCs are mostly absent in lymph under normal physiological conditions, RBC-derived EVs were reported to be present in lymphatic circulation (Milasan et al., 2016b). RBC-derived EVs measure between 100 and 300 nm (Arraud et al., 2014), contain hemoglobin and are surrounded by a lipid bilayer rich in acetylcholinesterase (Harisa et al., 2017). EVs derived from RBCs are associated with increased oxidative stress, including free heme transfer to endothelial cells (Camus et al., 2015), and thus they can interfere with NO signaling and promote initiation of endothelial dysfunction (Boulanger et al., 2017). They were also reported to scavenge NO faster than intact erythrocytes (Herring et al., 2013), causing vasoconstriction, increased erythrocyte adhesion and enhanced endothelial damage (Camus et al., 2015; Harisa et al., 2017).

Lymphatic contractions are tightly regulated by endothelium-derived relaxation factors such as NO (Gasheva et al., 2006) and histamine (Nizamutdinova et al., 2014). NO is released in a shear-dependent manner to limit contractions in periods of high lymph velocity while ensuring proper diastolic relaxation and lymphatic filling (Bohlen et al., 2011). However, NO bioavailability critically depends on the delicate balance between its production and degradation by reactive oxygen species (ROS). Disruption in this finely tuned balance can alter the lymphatic pumping and endothelial permeability (Zawieja et al., 1991; Gasheva et al., 2007).

Based on the findings stated above, RBC-derived EVs are likely to be involved in atherosclerosis-related lymphatic dysfunction given their capacity to increase oxidative stress and scavenge NO. Preliminary studies are in line with the results reported on the blood endothelium. RBC-derived EVs were found to increase oxidative stress on LEC and thus increase lymphatic endothelial permeability *in vitro* (Jean et al., 2018).

### Extracellular Vesicles Released by Platelets

Platelets are essential during embryogenesis (Uhrin et al., 2010a) and throughout life (Hess et al., 2014) in the maintenance of a proper lymphatic function, and Milasan et al. (2017) have reported that promising treatments in atherosclerosis are acting through platelet adhesion on LECs to exert their beneficial effects. Platelets regulate the blood/lymphatic vessel separation by inhibiting the proliferation, migration, and tube formation of LECs upon the interaction of C-type lectin-like receptor 2 (CLEC-2) with podoplanin (Osada et al., 2012). In the blood circulation, platelets have been shown to act through their secreted active releasates and extracellular vesicles (Arraud et al., 2014) to instigate (Massberg et al., 2002) and exacerbate (Huo et al., 2003) atherosclerosis. Albeit they do not form a normal constituent of lymph, our laboratory has demonstrated that PEVs are abundant in mouse lymph (Milasan et al., 2016b), and we now suspect that presence of these submicron particles in lymph may be critical in maintenance of a proper lymphatic function during atherosclerosis.

Platelets are versatile blood cells involved in thrombosis and hemostasis but are increasingly recognized as key players in innate and adaptive immune responses (Semple et al., 2011) as well as lymphatic vessel development (Hess et al., 2014). They are the major source of circulating EVs, releasing preferentially exosomes and MVs (Heijnen et al., 1999). PEVs have been extensively studied in various settings including vascular inflammation, atherosclerosis and hemostasis. The amount and cargo of PEVs are determined by whether their release is spontaneous or induced (Aatonen et al., 2014). They could be thus either beneficial or deleterious depending on the composition of the membrane and biological cargo contained in the vesicle. For instance, several studies suggest that PEVs increased blood endothelial permeability (Marcos-Ramiro et al., 2014; Edrissi et al., 2016; Boulanger et al., 2017). This effect could be due to enhanced apoptosis through delivery of caspase 3 from PEVs to ECs (Edrissi et al., 2016). However, a recent study reported that PEVs protect the microvasculature from factors, such as thrombin, capable of disrupting endothelial permeability (Miyazawa et al., 2019).

PEVs may also play a dual role in inflammation as they can induce either a pro- or anti-inflammatory response (Zaldivia et al., 2017). PEVs can activate the release of pro-inflammatory cytokines, including IL-1 and IL-6, and the expression of ICAM-1 by ECs (Barry et al., 1998). In the setting of atherosclerosis, PEVs activate ECs, promote monocyte adhesion and plaque recruitment. PLT-derived MVs can also contribute to atherogenesis by inducing hyperplasia of vascular SMCs (Weber et al., 2000). Alternatively, MVs shed by stored human platelets suppressed proinflammatory differentiation of monocytes to macrophages, as well as maturation of DCs (Sadallah et al., 2011). Another study has demonstrated that PLT-derived MVs hampered the differentiation of peripheral regulatory T cells into a pro-inflammatory phenotype (Dinkla et al., 2016). Moreover, PEVs are able to stimulate thrombosis (Mallat et al., 1999). Their procoagulant activity is mediated by the exposure of the anionic phospholipid PS or the expression of TF, main activator of the extrinsic pathway (Mackman et al., 2007).

Given their diverse biological effects, predicting the effect of PEVs on LECs seem quite challenging. However, platelets have been well recognized for their crucial role in lymphangiogenesis and lymphatic function throughout life (Suzuki-Inoue et al., 2007; Bertozzi et al., 2010; Hess et al., 2014). During embryogenesis, separation of lymphatic and blood circulation depends on platelet activation (Uhrin et al., 2010b). In fact, platelets prevent blood-lymphatic mixing at the lymphovenous junction by inhibiting the proliferation and migration of LECs, through CLEC-2/podoplanin interaction (Osada et al., 2012). Furthermore, a study has shown that platelets enhance the lymphatic endothelial integrity *in vitro* as they seem to exert a bridging effect between LECs (Milasan et al., 2017). Since platelets are absent from lymph, this protective effect could be mediated through PEVs. Preliminary work indicates that when PEVs are incubated on a lymphatic endothelium *in vitro*, they tend to be associated with a decrease in ROS production by LECs (Jean et al., 2018), which is known to cause cellular damage and alter DNA (Bhattacharyya et al., 2014). As opposed to RBC-derived EVs, PEVs could maintain lymphatic pumping by reducing the oxidative stress. Further deciphering the role of lymph PEVs (Milasan et al., 2016b) in the maintenance of lymphatic function and integrity would be of great interest.

### Extracellular Vesicles Released by Endothelial Cells

Blood ECs are an important source of EVs known to be involved in crosstalk between ECs, SMCs and immune cells in both normal and atherosclerotic conditions. EC-derived EVs are released in response to extracellular stimuli that trigger changes in phenotype and tissue remodeling (Danielson and Das, 2014). Similar to other cell types, they contain a multitude of proteins and RNAs (de Jong et al., 2012). Various stressful conditions such as hypoxia or tumor necrosis factor alpha (TNF-α), both simulating an inflammatory environment as observed in atherosclerosis, affected the proteome and transcriptome of EVs secreted by cultured ECs (de Jong et al., 2012; Chistiakov et al., 2015). In hypoxic EVs, proteins involved in stress response and proapoptotic function were observed (Melotte et al., 2010). In TNF-α induced EVs, significant changes in the amount of mRNA were observed, especially proinflammatory ones such as IL-8, MCP-1, IL-32, and VCAM-1 (Chistiakov et al., 2015). In another study, EVs released by cultured serum-starved human ECs that underwent advanced apoptosis and autophagy were shown to contain autophagosomes, as well as mitochondria, and delivered various danger signals including ATP release which is involved in autophagy regulation (Pallet et al., 2013). As such, detection of these EC-derived apoptotic EVs in blood may suggest endothelial dysfunction (Chistiakov et al., 2015).

Exposure to higher shear stress is another factor that predisposes to atherosclerosis, and in human umbilical vein endothelial cells, leads to formation of EC-derived EVs that contain miR-143/145 clusters (Boon and Horrevoets, 2009). The latter were shown to prevent hyperplasia and maintain the contractile phenotype of co-cultured SMCs (Cheng et al., 2009; Hergenreider et al., 2012).

Taken together, all these findings allow us to consider LECs-derived EVs as an attractive new tool to assess lymphatic vessel function. LECs are now known to also be active players in the production of EVs. Recent findings suggest that LECs *per se* can release exosome-rich endothelial vesicles to guide the migration of cancer cells and promote their metastasis, a phenomenon that was enhanced after exposure to the inflammatory cytokine TNFα (Brown et al., 2018). While lymphatic exosomes appear to aid with cellular transport through the lymphatics, all types of LEC-derived EVs can also be used as biological particles reflective of lymphatic integrity. Since lymphatic dysfunction occurs even before the onset of atherosclerosis (Milasan et al., 2016a), characterizing LECs-derived EVs in blood circulation could potentially become an early diagnostic tool of lymphatic dysfunction, while assessing the risk of atherosclerosis.

## Concluding Remarks

Extracellular vesicles and lymphatic vessel function are emerging biomarkers for the prevention of atherosclerosis. Whereas past studies focused on role EVs in the onset and progression of atherosclerosis, the interaction between EVs and the lymphatic system during atherosclerosis is understudied. We herein sought to concatenate evidence that EVs could be at the interplay between lymphatic function and atherosclerosis. The field of EVs is quickly growing, however, several factors must be considered when assessing their function. One major necessity in the field is to improve and standardize methods for EVs isolation and analysis (Thery et al., 2006). Their small size adds up to the challenge of performing lymph puncture and complexifies the proper identification of the specific EVs subtypes with the imaging tools available to date. A multitude of isolation methods that produce distinct populations of EVs do exist, and new ones emerge relatively often, which makes data comparability difficult. Once EVs have been isolated from their respective media, the biggest problem to date remains EVs purity, which is crucial when evaluating EVs dosage for functional studies and efficient therapies (Xu et al., 2016). Currently, no method allows an entire and accurate phenotyping, characterization and sizing of all types of EVs. Thus, the EVs community acknowledges the need for a standardized, feasible and cost-effective method to isolate and analyze EVs properly. With emerging research, some consensus has been achieved and continues to evolve (van der Vorst et al., 2018). The International Society for Extracellular Vesicles (ISEV) attempts to provide clear guidelines in an effort to standardize EVs procedures (Thery et al., 2018). To further improve reliability, the EV-TRACK (transparent reporting and centralizing knowledge in EV research) platform was developed (Consortium et al., 2017; van der Vorst et al., 2018). Its purpose is to encourage scientists to integrate all collected data in a uniform matter with associated details so that studies can be accurately replicated and compared (van der Vorst et al., 2018). The involvement of EVs in chronic inflammatory diseases such as atherosclerosis is reputable. Refining and standardizing the characterization of these small vesicles in lymph will enable the discovery of new prognostic markers bridging the onset and progression of several pathologies to an impairment in lymphatic function.

## Author Contributions

AM, MF, and CM contributed to the concepts, writing, and editing of the manuscript.

## Conflict of Interest

The authors declare that the research was conducted in the absence of any commercial or financial relationships that could be construed as a potential conflict of interest.
